# Comparison of Cavum Septum Pellucidum Size in Euploid and Aneuploid Fetuses

**DOI:** 10.1055/s-0043-1775847

**Published:** 2023-10-16

**Authors:** Merve Ozturk Agaoglu, Zahid Agaoglu, Filiz Halici Ozturk, Sevki Celen, Turhan Caglar

**Affiliations:** 1 Department of Perinatology, Turkish Ministry of Health Ankara City Hospital, Ankara, Turkey.; 2 Department of Perinatology, Etlik City Hospital, Ankara, Turkey.

**Keywords:** aneuploidy, cavum septum pellucidum, karyotype, aneuploidia, cariótipo, septo do cavum pelúcido

## Abstract

**Objective** The aim of the present study is to compare the cavum septum pellucidi (CSP) z-score in euploid and aneuploid fetuses and to investigate the performance of the CSP width/length and CSP width/biparietal diameter (BPD) ratios as a diagnostic marker in aneuploidy.

**Methods** A total of 54 patients, 20 aneuploid and 35 euploid fetuses, between 18 and 37 weeks of gestation, were included in this retrospective study. The CSP width z-score was compared between the two groups. Receiver operating characteristic (ROC) curves were calculated for the CSP width/length and CSP width/BPD ratios to predict aneuploidy.

**Results** The median CSP width was 4.8 mm (range, 1.8 to 8.5 mm) in the euploid group, and 5.4 mm (range 3.1 to 8.4 mm) in the aneuploid group. Cavum septum pellucidi width z-score, CSP width/length ratio, and CSP width/BPD ratio were significantly higher in fetuses with aneuploidy than in fetuses with normal karyotype ( *p* = 0.001; *p* = 0.013; *p* = 0.028). In the ROC analysis, the CSP width/length ratio had the optimal cutoff value of 0.59, with 72.0% sensitivity and 58.0% specificity, and for the CSP width/BPD ratio, the cutoff value was 0.081 with 83.0% sensitivity and 61.0% specificity for detection of aneuploidy.

**Conclusion** CSP width z-score was found to be increased in aneuploid fetuses. The CSP width /BPD ratio can be used as a new marker for predicting aneuploidy.

## Resumo

**Objetivo:** O objetivo do presente estudo é comparar o escore z do cavum septum pellucidi (CSP) em fetos euploides e aneuploides e investigar o desempenho das relações largura/comprimento do CSP e largura do CSP/diâmetro biparietal (BPD) como marcador diagnóstico de aneuploidia. como marcador de diagnóstico de aneuploidia.

**Métodos:** Um total de 54 pacientes, 20 fetos aneuploides e 35 fetos euploides, entre 18 e 37 semanas de gestação, foram incluídos neste estudo retrospectivo. O escore z da largura da CSP foi comparado entre os dois grupos. As curvas ROC (Receiver Operating Characteristic) foram calculadas para as relações largura/comprimento da PEC e largura da PEC/BPD para prever a aneuploidia.

**Resultados:** A largura mediana da CSP foi de 4,8 mm (variação de 1,8 a 8,5 mm) no grupo euploide e de 5,4 mm (variação de 3,1 a 8,4 mm) no grupo aneuploide. O escore z da largura do cavum septum pellucidi, a relação largura/comprimento do CSP e a relação largura do CSP/BPD foram significativamente maiores em fetos com aneuploidia do que em fetos com cariótipo normal ( *p* < 0,001; *p* < 0,013; *p* < 0,028). Na análise ROC, a relação largura/comprimento da CSP teve o valor de corte ideal de 0,59, com 72,0% de sensibilidade e 58,0% de especificidade, e para a relação largura da CSP/BPD, o valor de corte foi de 0,081, com 83,0% de sensibilidade e 61,0% de especificidade para a detecção de aneuploidia.

**Conclusão:** Verificou-se que o escore z da largura da CSP estava aumentado em fetos aneuploides. A relação A relação largura da CSP /BPD pode ser usada como um novo marcador para prever a aneuploidia.

## Introduction

The cavum septum pellucidum (CSP) is an intracranial structure on axial images of the fetal head examined during routine obstetric examinations as recommended in the guidelines for fetal neurosonography. 1 2 It is a hypoechoic, roughly rectangular formation in the midline, in front of the third ventricle. The CSP can be detected between 16 weeks and birth. Although it resolves after delivery when the 2 lamellae of the cavum form the septum pellucidum, it is observed in the majority of preterm newborns and in around 50% of full-term infants. 3 4 5 In the studies performed, the absence of CSP has been highlighted as an indication of the presence of several midline abnormalities, including corpus callosum dysgenesis, holoprosencephaly, and agenesis of the septum pellucidum. 6 7 8

Routine anatomical screening recommended to detect aneuploidy in the 2 ^nd^ trimester mainly includes autosomal trisomies and triploidy. The most common aneuploidies are trisomy 21, 18, 13, and triploids. Although there are specific findings for each trisomy, cardiovascular and cranial system anomalies are the most common system anomalies. 9 10 11 12 Recently, it has been noted that CSP tends to be larger than normal in fetuses with chromosomal abnormalities, and studies have been conducted on CSP measures associated with fetuses with trisomy 18, 21, and 13. 13 However, the results are inconclusive because there are not enough studies in this area.

Our study aimed to compare the size of CSP in euploid and aneuploid fetuses and to investigate the ratio of CSP width/length and CSP width/biparietal diameter (BPD) in both groups.

## Methods

A total of 54 patients, 20 aneuploid and 34 euploid fetuses, admitted to the perinatology outpatient clinic of the tertiary center between May 2019 and September 2019 were included in the study. The stored ultrasound data of fetuses between 18 and 37 weeks were retrospectively analyzed. All patients gave informed consent for ultrasound examination and consented to digital data storage. The hospital ethics committee approved the study. The study was conducted in accordance with the Declaration of Helsinki.

We searched the databases of our center for pregnancies diagnosed with prenatal trisomy 21, 18, triploidy, or Turner syndrome by amniocentesis or chorionic villus sampling and who underwent ultrasonography after 20 weeks of gestation. Pregnancies known to result in neonates with normal outcomes and aneuploid pregnancies between 20 and 37 weeks of gestation were included in our analysis.

Patients were included in the study if the proximal and distal hemispheres were seen in the same size cross-section of the fetal head, the standard anatomic points required to measure the fetal head, and the image containing the CSP. Other inclusion criteria were pregnancies in which an ultrasound examination was performed in the 1 ^st^ trimester. Twin pregnancies, fetal growth restriction, anomalies with the absence of CSP, such as holoprosencephaly or agenesis of the corpus callosum, or fetuses with other cranial anomalies were excluded from the analysis. All ultrasound examinations were performed with a GE Voluson E8 convex transabdominal probe (1.75 to 4.95 MHz), and measurements were made on the acquired images and stored in the database.

**Fig. 1:**
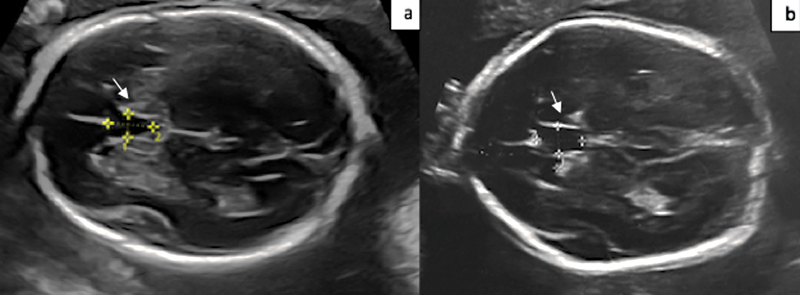
Two-dimensional ultrasound images of the cavum septi pellucidi (white arrow) in a euploid fetus (a) and in a fetus with trisomy 18 (b).

Maternal characteristics, gestational age at the time of examination, BPD (mm), occipitofrontal diameter (mm), size of CSP (mm), fetal karyotype, and lateral ventricle measurement (mm) were recorded in the control and case groups. The length and width of CSP were measured on images stored in the patient registry, and the CSP width was measured at the center of CSP as described by Abele et al. 13 The CSP length was measured between the callosal sulcus anteriorly and the fornix posteriorly ( Fig. 1 ).

The measurement of the width of CSP was converted to a z-score according to the table defined by Zhou et al. so that it was not affected by gestational week. 14 The CSP ratio was calculated by dividing the width of the CSP by its length, and then the other ratio, the CSP width/BPD ratio, was calculated. The two groups were compared based on maternal characteristics, CSP size, and the associated ratios. IBM SPSS Statistics for MAC, version 22.0 (IBM Corp., Armonk, NY, USA) was used to perform the statistical analyses. Frequency tables and descriptive statistics were used to analyze the results. Visual (histograms, probability plots) and analytical methods were used to determine whether the variables were normally distributed or not. For the nonparametric distribution, the Mann-Whitney U test was used to compare the groups. The Spearman correlation coefficient was used to examine the relationship between groups that did not have a normal distribution. Receiver operating characteristic (ROC) analysis was used to assess the predictive performance of the CSP width/length and of the CSP width/BPD ratio for aneuploidy. P-values < 0.05 were considered statistically significant.

## Results

The present study included 54 pregnancies, 34 with euploid fetuses, and 20 aneuploid fetuses. There were 9 (45%) fetuses with trisomy 21, 8 (40%) fetuses with trisomy 18, 2 (10%) fetuses with triploidy, and 1 (5%) fetus with Turner syndrome. In the aneuploid group, the median maternal age was 36, significantly higher than in the euploid group (30 versus 36; *p* = 0.004). The median gestational age at ultrasound examination was similar for both groups (27 versus 23.3; *p* = 0.771) ( Table 1 ).

**Table 1:** Maternal and fetal ultrasound characteristic in the euploid and aneuploid groups

	Euploid group ( *n* = 34)	Aneuploid group ( *n* = 20)	*p-value*
Age, years old	30 (24–40)	36 (26–45)	0.004
Gravida	3 (1–6)	2.5 (1–5)	0.558
Parity	1 (0–3)	1.5 (0–3)	0.124
Gestational age at ultrasound examination, week	27 (20–34)	23.3 (20–34)	0.771
BPD (mm)	67.0 (47–84)	58.7 (45–91)	0.100
HC (mm)	248 (168–302)	214 (161–329)	0.265
OFD (mm)	89 ( 58–108)	75.9 (53–115)	0.168

Abbreviations: BPD, biparietal diameter; HC, head circumference; OFD, occipitofrontal diameter.

Data shown as median (min-max).

*p* < 0.05 is considered statistically significant.

The median CSP width was 4.8 mm (range, 1.8 to 8.5 mm) in the euploid group, and 5.4 mm (range 3.1 to 8.4 mm) in the aneuploid group. A statistical difference was detected between euploid and aneuploid groups by terms of CSP width z-score ( *p* = 0.001) ( Table 2 ).

**Table 2:** Comparison of CSP width and CSP ratios in euploid and aneuploid groups

	Euploid group ( *n* = 34)	Aneuploid group ( *n* = 20)	*p-value*
CSP width (mm)	4.8 (2.3–8.5)	5.4 (3.1–8.4)	0.173
CSP width Z-score	- 0.5 (-2.0–2.0)	1.0 (- 1.0–3.0)	0.001
CSP length (mm)	9.0 (5–12)	8.7 (5–15)	0.589
CSP width/CSP length	0.57 (0.32–0.85)	0.62(0.49–0.88)	0.013
CSP width/BPD (mm)	0.080 (0.05–0.11)	0.088 (0.06–0.15)	0.028
Lateral ventricule (mm)	5.8 (3.5–8.0)	6.6 (5.0–9.6)	0.045

Abbreviations: BPD, biparietal diameter; CSP, cavum septum pellucidi.

Data shown as median (min-max).

*p* < 0.05 is considered statistically significant.

**Fig. 2:**
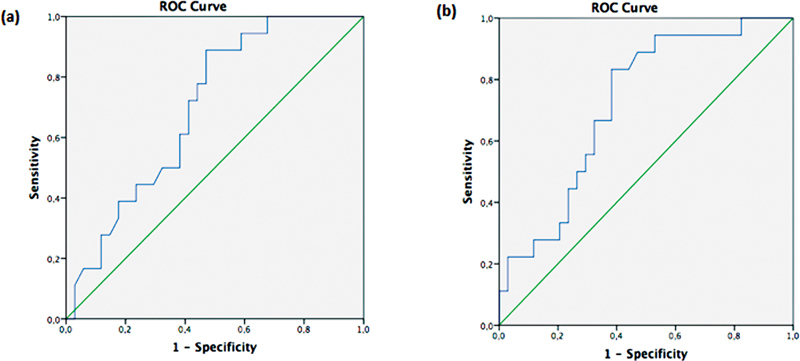
ROC curve of CSP width/length (a) and CSP width/BPD ratio (b) to differentiate aneuploidy.

A significantly lower CSP z-score was found in fetuses with aneuploidy than in the control group. In the fetuses with trisomy 21, 18, and triploidy, the median CSP width was 4.8 mm (range 3.1 to 7.5 mm), 6.9 mm (range 4.8 to 8.4 mm), and 5.0 mm (range 4.8 to 5.2 mm), respectively. The CSP width/length ratio and the CSP width/BPD ratio was higher in fetuses with aneuploidy compared with fetuses with normal karyotype ( *p* = 0.013; *p* = 0.028). In the ROC analysis, the CSP width/length ratio had the optimal cutoff value of 0.59, with 72.0% sensitivity and 58.0% specificity, and for the CSP width/BPD ratio, the cutoff value was 0.081, with 83.0% sensitivity and 61.0% specificity for detection of aneuploidy ( Table 3 ) ( Fig. 2 ).

**Table 3:** Diagnostic values of CSP width/length, CSP width/BPD ratios to differentiate aneuploidy

Value	CSP width/length	CSP width/BPD
Cutoff	0.59	0.081
Area under the receiver operating curve	0.69	0.72
Sensitivity	72	83
Specificity	58	61

Abbreviations: BPD, biparietal diameter; CSP, cavum septum pellucidi.

In the aneuploid group, a significant strong positive correlation was found between CSP width z-score and CSP width /BPD ratio ( *p* = 0.00; *r* = 0.875). In contrast, the CSP width z-score did not correlate with the CSP width/length ratio in the aneuploid group ( *p* = 0.302).

## Discussion

In the present study, we investigated CSP z-score, and CSP ratios in aneuploid fetuses as a diagnostic tool to distinguish aneuploid fetuses. We found a higher CSP z-score in aneuploid fetuses compared with the euploid group. Moreover, the CSP width/BPD ratio, a new marker, was significantly higher in aneuploid fetuses.

The International Society of Ultrasound in Obstetrics and Gynecology recommendations for baseline evaluation of the central nervous system include the visualization of the CSP. 1 2 The CSP serves as a reference for establishing the proper axial plane when evaluating BPD. Previous research has established reference ranges for CSP width. 13 14 15 16 Large CSP has been recognized as a marker for fetal neural developmental abnormalities, often affecting the septohippocampal and limbic systems. 13 Several studies have shown that a CSP of > 1 cm is an important indicator of neurological impairment and may be associated with an increased risk of cognitive delays and behavioral problems. 17 18 Abele et al reported that the width of CSP due to chromosomal abnormalities was large, particularly in trisomy 18. 13 The CSP was found to be enlarged in 92, 40, and 41% of fetuses with trisomy 18, 13, and 21, respectively. In another study, Chaoui et al. investigated the relationship between del.22q11 and CSP size and found that 67.5% of fetuses with del.22q11 had an enlarged CSP. 19 Based on the publication on the relationship between the size of the CSP and genetic abnormalities, we investigated CSP size in aneuploid fetuses and determined a new ratio that is independent of head size. Our study revealed that the CSP width /BPD ratio can help identify aneuploid fetuses, with higher specificity than the CSP width/length ratio in aneuploid fetuses. The pathophysiological mechanism of CSP enlargement in fetuses with aneuploidy is not clearly defined. Considering that larger lateral ventricles are usually found in aneuploid fetuses, cerebrospinal fluid filling of the CSP by the anterior horns of the lateral ventricles could explain the enlargement of the CSP associated with fetal aneuploidy. 19 In another study, it was suggested that it might be caused by an abnormality in one of the surrounding structures of the CSP or by an abnormality causing increased diffusion of cerebrospinal fluid within the septum pellucidum. 13 The relationship between CSP size and intracranial structures has been discussed in many recent studies. Shen et al. reported that in partial agenesis of the corpus callosum, CSP was enlarged and the width of the CSP is greater than its length. 20 Karl et al. also defined the CSP length/width as a CSP ratio and found that < 1.5 was associated with partial corpus callosum agenesis. 21 They concluded that the CSP ratio has the potential to identify fetuses at high risk for partial corpus callosum agenesis. In our study, we found that a CSP width/length ratio > 0.59 was significant in determining aneuploid fetuses. A recent study examined the efficacy of the ratio of CSP width to anterior-posterior cerebellar diameter (APCD) as a diagnostic tool for prenatal trisomy 18 diagnosis. 22 A higher CSP/APCD ratio would support the diagnosis, especially in cases with trisomy 18 syndrome with few abnormalities.

The strength of our study is that we have presented the CSP width/BPD ratio, a new practical and gestational week-independent marker to predict aneuploidy. The limitation of our study is its retrospective design. Because of the retrospective design, we were unable to follow-up and evaluate the size of the CSP in the neonatal period.

## Conclusion

It is important to examine the dimensions of the CSP and its relationship to other structures as part of basic prenatal screening. An easily measurable abnormal CSP width/length ratio and the more specific CSP width/BPD ratio can serve as a simple indicator for the possible presence of aneuploidy. To the best of our knowledge, this is the first study to address CSP width/BPD ratio in aneuploid fetuses. Although previous studies show that CSP is increased in aneuploid fetuses, prospective studies are needed to demonstrate the applicability of the CSP width/BPD ratio for predicting aneuploidy in clinical practice.
